# Ten-Year Persistence of Biologic Drugs in Psoriasis and Its Relationship with Pharmacogenetic Biomarkers

**DOI:** 10.3390/biomedicines13010005

**Published:** 2024-12-24

**Authors:** Andrea Rodríguez-Lopez, María Martínez-Sendino, Rocío Prieto-Pérez, Paula Soria-Chacartegui, Eva González-Iglesias, Mario Aparicio-Domínguez, Sonsoles Berenguer-Ruiz, Esteban Daudén, Francisco Abad-Santos

**Affiliations:** 1 Clinical Pharmacology Department, Hospital Universitario de La Princesa, Faculty of Medicine, Universidad Autónoma de Madrid (UAM), Instituto de Investigación Sanitaria La Princesa (IP), 28006 Madrid, Spain; arodriguezl.externo@salud.madrid.org (A.R.-L.); mariamtzsendino@gmail.com (M.M.-S.); egiglesias@salud.madrid.org (E.G.-I.); 2 Clinical Pharmacology Department, Hospital Universitario La Paz, Department of Pharmacology and Therapeutics, Faculty of Medicine, Universidad Autónoma de Madrid (UAM), IdiPAZ, 28046 Madrid, Spain; rociomaria.prieto@gmail.com; 3 Dermatology Department, Hospital Universitario de La Princesa, Instituto de Investigación Sanitaria La Princesa (IP), 28006 Madrid, Spain; mario.aparicio@salud.madrid.org (M.A.-D.); sonsoles.berenguer@salud.madrid.org (S.B.-R.); esteban.dauden@salud.madrid.org (E.D.); 4 Centro de Investigación Biomédica en Red de Enfermedades Hepáticas y Digestivas (CIBERehd), Instituto de Salud Carlos III, 28029 Madrid, Spain

**Keywords:** psoriasis, persistence, biologic therapies, loss of efficacy, pharmacogenetic biomarkers

## Abstract

**Background**: Psoriasis is a skin disease characterized by the presence of erythematous, scaly plaques on the extensor surfaces of the body. Treatment varies according to the stage of the disease, with the most severe cases being treated with biologic treatments that differ in efficacy and persistence over time. This study aimed to evaluate the 10-year persistence of biologic drugs (adalimumab, etanercept, infliximab and ustekinumab) in the treatment of moderate-to-severe plaque psoriasis. **Methods**: A total of 143 patients (61 women and 82 men) were evaluated; data were collected from the electronic clinical history, and statistical analysis was performed using the SPSS program. In addition, 115 of them were genotyped in a previous study for 173 immune system genetic polymorphisms. **Results**: The persistence of biologic drugs at 10 years was 25.9% (95% CI: 17.2–34.5%). Adalimumab was the most persistent drug (41.5%), followed by ustekinumab (34.8%), infliximab (28%) and etanercept (9.3%). The main reason for discontinuation was insufficient efficacy (51%). Adalimumab allowed an increase in the dosing interval in 82.4% of patients who persisted and ustekinumab allowed an increase in 37.5%. The 10-year persistence was related to sex (higher in men, *p* < 0.001), biologic drug (*p* = 0.002) and polymorphisms in *LMO4* (rs983332) (*p* = 0.014) and *IL20RA* (rs1167846) (*p* = 0.013). **Conclusion**: The results show that 25% of psoriasis patients treated with first-line biologics persisted at 10 years.

## 1. Introduction

Psoriasis is a chronic immune-mediated inflammatory disease of the skin. It is characterized by the formation of erythematous and scaly plaques on various parts of the body, although they tend to occur in areas of extension (elbows, knees, skull or lumbar area of the back) [1]. Its current prevalence ranges between 0.14% and 1.99% [2], making it a common disease in the population. In cases of mild psoriasis, treatment consists of topical medications and phototherapy, while as the disease progresses, systemic therapy is initiated to reduce symptoms and improve the patient’s quality of life [3]. It is a disease that can also be conditioned by external factors such as smoking, alcohol or stress [2]. For patients with moderate-to-severe psoriasis, systemic biologic agents targeting tumor necrosis factor-alpha (TNF-alpha) and interleukin 12/23 (IL-12/23) were the first therapies to transform disease management [4]. However, recent advances in understanding the pathophysiology of psoriasis have paved the way for the development of newer treatments, such as anti-interleukin 17 and anti-interleukin 23 agents, which have further expanded the therapeutic options available.

As a chronic disease that requires continuous treatment over time, the persistence of therapy has been shown to be affected by several factors. Discontinuation of treatment may be due to loss of efficacy over time [5,6], loss of the follow-up, adverse effects associated with each drug or the need to switch to another treatment [7], to increase the dose of the current drug or to add another drug [8].

In 2010, a research project (PI10/01740) entitled “Search for genetic markers predictive of response to biologic drugs in the treatment of psoriasis” was conducted by the Clinical Pharmacology and Dermatology Services of the Hospital Universitario de La Princesa. The aim of this project was to determine the genetic polymorphisms that can predict the therapeutic response to biologic drugs in order to be able to select those patients who will respond favorably to treatment. It was planned to enroll 180 patients with moderate-to-severe psoriasis who were being treated with biologic agents according to standard clinical practice. This study was approved by the Clinical Research Ethics Committee (CEIC) on 22 April 2010 (code PI-445) and enrolled 172 patients to evaluate the efficacy of the biologic agents administered for psoriasis in those years (anti-TNF-alpha: adalimumab, infliximab and etanercept and anti-IL-12/23: ustekinumab) [4].

This is an extension of the previous study [4], where single-nucleotide polymorphisms (SNPs) that can predict the response to anti-TNF drugs in Caucasian patients with moderate-to-severe plaque psoriasis were investigated. This study aimed to evaluate the 10-year persistence of treatment with biologic agents for moderate-to-severe psoriasis, persistence being understood as the continuity of treatment without interruption. In addition, the reasons for discontinuation and the number of patients who had to increase or decrease the drug dose were examined. Moreover, the effect of genetic variation on treatment persistence at 10 years was studied. To the best of our knowledge, this is the first article to evaluate the persistence of all these drugs at 10 years.

## 2. Materials and Methods

### 2.1. Study Population

A retrospective observational study was conducted by collecting data from the medical records of patients treated with this biologic therapy for psoriasis at the Hospital Universitario de La Princesa who participated in a previous study conducted at the same hospital in 2010–2013. This study was approved on 13 December 2023, by the Ethics Committee for Drug Research (CEIm) of the Hospital Universitario de la Princesa (registry number 5432).

The inclusion criteria included a diagnosis of moderate-to-severe psoriasis, being treated at the Hospital Universitario de La Princesa, being treated with biologic drugs, starting the treatment before the end of 2013 and giving consent to participate in the psoriasis pharmacogenetics study PI10/01740. The exclusion criteria included not having a clinical history available in the Hospital Universitario de La Princesa platform.

The following data were collected from the medical records: age, sex, date of diagnosis, current diagnosis, medication received, date of treatment initiation and discontinuation, reasons for discontinuation, PASI (Psoriasis Area Severity Index) at initiation and discontinuation, other comorbidities and smoking. Persistence was considered when the same drug was received continuously for 10 years, although temporary interruptions were allowed for reasons such as pregnancy or surgery. All information was collected during January 2024. This information was obtained only from the database of the hospital computer system, without the need to interview patients or obtain their informed consent to obtain additional information. The work consisted of reviewing the medical records without causing any change in the information system or altering routine clinical practice. Only coded clinical data were used to ensure confidentiality.

### 2.2. Genotyping

The starting point was the data obtained in the previous work by Prieto-Pérez R., in which 173 polymorphisms were evaluated using the Illumina Veracode genotyping platform (Human Genotyping Unit-CeGen, Madrid, Spain). A description of the SNPs studied is shown in Supplementary Table S1 published by Prieto-Pérez et al. [9].

### 2.3. Statistical Analysis

The main study variables were the percentage of treatment persistence at 10 years, defined as patients still receiving the first prescribed biologic, and the effect of genetic variation on treatment persistence at 10 years.

The secondary variables analyzed were the age at diagnosis of psoriasis, the age at treatment initiation, sex, PASI at treatment initiation, PASI at treatment discontinuation, date of treatment discontinuation, concomitant medications, adverse effects and reasons for switching.

The IBM SPSS Statistics (version 23, SPSS Inc., Chicago, IL, USA) software was used for statistical analysis. In order to analyze quantitative variables in terms of categories (sex, drug administered and 10 year-persistence), the *t*-test or ANOVA was employed. Similarly, for the analysis of qualitative variables in terms of these categories, the chi-square test was utilized. Multivariate analyses were performed using binary logistic regression (LR), including those independent variables that were significantly associated with the dependent variable in the univariate analysis (i.e., with univariate *p*-values (*p*_uv_) lower than 0.05). The method used was forward LR and the results were expressed as the odds ratio (OR) with the 95% confidence interval (CI).

## 3. Results

### 3.1. Population

**Figure 1:**
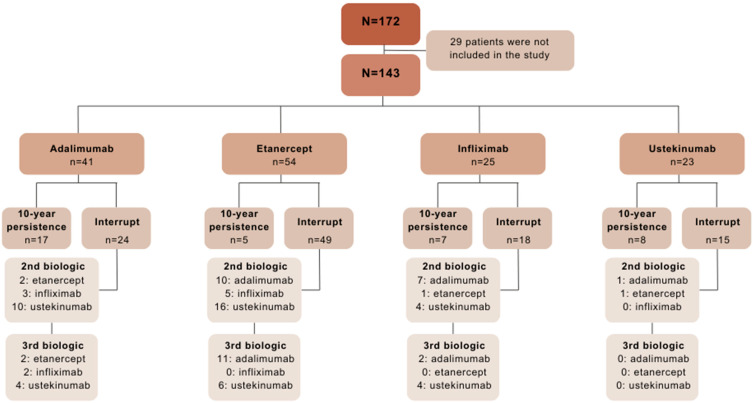
Flow-chart of patients initiating treatment with biologics for psoriasis according to the drug prescribed as first-line treatment and showing the second and third options in patients who discontinued. Of the patients that interrupted their treatment, only those who switched to one of the other three alternative drugs in this research are shown.

Of a total of 172 initial patients, 29 patients were excluded for various reasons. The most common reason was loss of the follow-up before 2013 (*n* = 10), followed by lack of biologic treatment (*n* = 6), administration of efalizumab prior to the biologics studied (*n* = 5), no physical medical history (*n* = 5) and no diagnosis of psoriasis (*n* = 3). Finally, 143 patients who met the inclusion criteria were studied (Figure 1).

Table 1 shows the characteristics of the 143 patients, 61 women and 82 men, with a mean age of 44.7 ± 15.1 years at the start of biological treatment. Psoriatic arthritis was present in 38.5% of patients. A total of 32 patients (22.4%) were identified as smokers, with a higher prevalence observed among men (28%) compared to women (14.8%), although this did not reach statistical significance. Regarding cardiovascular risk factors (hypertension, dyslipidemia and diabetes mellitus), 91 patients (63.6%) had one of these three risk factors assessed. In total, 41 patients received adalimumab, 54 etanercept, 25 infliximab and 23 ustekinumab as first-line treatment. The distribution of drugs was similar between the sexes, except for etanercept, which was used more often in women (*p* = 0.023) (Table 1). Many patients received these drugs as second- and third-line therapy (Figure 1).

The doses of the drugs administered were those indicated in the drug label. For adalimumab, an initial dose of 80 mg followed by 40 mg subcutaneously every 2 weeks. For etanercept, 50 mg subcutaneously every week. For infliximab, 5 mg/kg intravenously at baseline, 2 weeks, and 6 weeks and then every 8 weeks. For ustekinumab, 45 mg subcutaneously followed by 45 mg at 4 weeks and then 45 mg every 12 weeks for patients weighing <100 kg and 90 mg at baseline and 4 weeks and then 90 mg every 12 weeks for patients weighing >100 kg.

Persistence at 10 years after initial treatment was 25.9% (95% confidence interval, 95% CI: 17.2–34.5%) (see Table 2). Among those patients treated with adalimumab, 41.5% persisted (95% CI: 31.8–51.0%) and 51.2% discontinued due to an insufficient efficacy (according to the criteria of the doctor and the patient). Among those treated with etanercept, 9.3% (95% CI: 3.4–15.2%) persisted. This drug was discontinued due to insufficient efficacy in 57.4% of patients. In this case, there were 5 patients (9.3%) who discontinued due to the appearance of adverse reactions and 2 patients discontinued due to a good response to treatment. As for infliximab, 28% continued receiving the treatment (95% CI: 19.2–36.8%). The main reason for discontinuation was insufficient efficacy in 52%. Finally, ustekinumab was also considered as a first-line treatment in 23 patients, and 34.8% (95% CI: 25.6–44.0%) persisted, 34.8% discontinued due to insufficient efficacy and 8.7% discontinued due to lack of control of psoriatic arthritis.

The 10-year persistence was compared between the four drugs evaluated as first-line treatments (Table 3), and a statistically significant result was obtained (*p* = 0.002), indicating that persistence was higher with adalimumab and lower with etanercept (Table 3). For the other factors, no differences were found between the four treatment groups (Table 3). Insufficient efficacy was the most common reason for discontinuation, with a total percentage of 62.9% of the 116 patients who discontinued, with no significant differences between the four treatments (*p* = 0.096), although the percentage was lower for ustekinumab. The adverse reactions that became the reason for discontinuation in etanercept treatment were guttate psoriasis in three patients, disabling diarrhea in one patient and thrombophlebitis and pneumonia in one patient. In adalimumab treatment, the only reason for discontinuation was erythroderma in one patient. During infliximab infusion, nausea, malaise and vomiting were present in two patients, which led to discontinuation.

Patients who remained on any of the first-line drugs were also considered for dose intensification or dose reduction based on a good response. Of the 17 patients who remained on adalimumab as first-line therapy, 1 (5.9%) had the dose increased to 80 mg every 2 weeks, while 14 (82.4%) of them had the dosing interval extended as follows: every 3 weeks in 5 patients, every 4 weeks in 7 patients, every 5 weeks in 1 patient, and every 16 weeks in 1 patient. For infliximab, the dose was increased in one (14.3%) of the seven patients who remained on infliximab, from 400 mg every 8 weeks to 450 mg every 8 weeks. Regarding etanercept, the dose was increased to 75 mg every week in one of the five patients (20%) who persisted. Finally, of the eight patients who persisted on ustekinumab, one patient (12.5%) <100 kg ended up on an increased dose of 90 mg every 12 weeks and three patients (37.5%) had the dosing interval extended to every 18 weeks, every 16 weeks and every 48 weeks. In the remaining patients that persisted, neither the dose nor the dosing interval was modified. Therefore, adalimumab was the drug in which de-intensification was more frequent, followed by ustekinumab, whereas in infliximab and etanercept, no de-intensification was conducted (*p* = 0.006) (Table 3).

Table 4 compares patients who persisted on the first drug with those who did not. Of the characteristics described, there was less persistence in women: 6 of 61 (9.8%) persisted compared to 31 of 82 (37.8%) of men (*p* < 0.001). There was also a tendency for patients with psoriatic arthritis, smokers, hypertensives, dyslipidemics and diabetics to persist less, but none of these factors reached statistical significance (Table 4).

**Figure 2:**
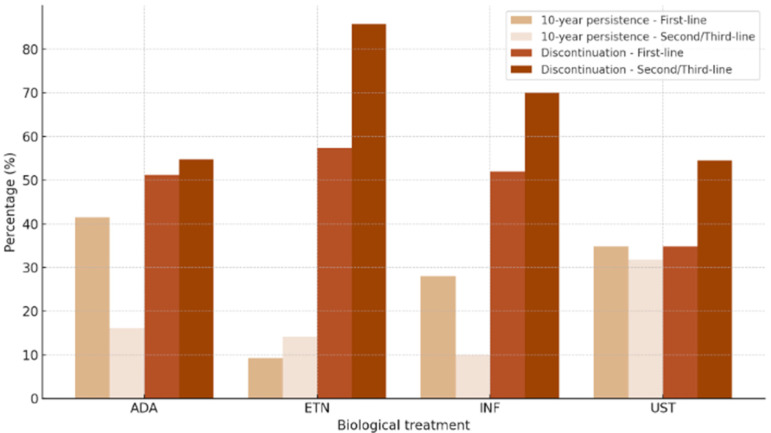
The 10-year persistence and discontinuation by insufficient efficacy according to prescription of biologics drugs as first-line or second- and third-line options. ADA: adalimumab; ETN: etanercept; INF: infliximab; UST: ustekinumab.

If the 10-year persistence was compared according to the administration of the first-line option or second- and third-line option, it was higher for the first-line treatment, although it only reached statistical significance for adalimumab (*p* < 0.021) (Figure 2), so persistence was lower when it was administered as a second- or third-line option because these were usually patients who had not responded to previous treatments. Moreover, discontinuation by insufficient efficacy was higher on second- and third-line treatments (Figure 2), with a statistically significant difference for etanercept (*p* < 0.003). In Supplementary Tables S1–S4, this information is shown according to biologic treatment and sex, with no significant differences between men and women.

### 3.2. Current Treatment

The treatment received by the patients in the study in January 2024 was also taken into account, where it was observed that 61 patients (42.7%) were treated with classical biologics such as adalimumab (*n* = 23), infliximab (*n* = 7), ustekinumab (*n* = 24) and etanercept (*n* = 7). In January 2024, 33.6% of patients (48 of 143) were being treated with other newer biologics for psoriasis, such as secukinumab (*n* = 7), ixekizumab (*n* = 6), risankizumab (*n* = 8), brodalimumab (*n* = 4), certolizumab (*n* = 5) and guselkumab (*n* = 18). There were two patients treated with methotrexate and nine patients treated with phototherapy because their disease had improved and they no longer needed systemic treatment. On the other hand, 23 patients were lost during their follow-up or had no current treatment in their history.

### 3.3. Genetic Factors

There were significant differences in 10-year persistence according to the genotype of the single-nucleotide polysmorphisms (SNPs) *LMO4* rs983332 and *IL20RA* rs1342642, rs1167846 and rs1184860 (Table 5). However, only the differences in *LMO4* rs983332 and in *IL20RA* rs1167846 were maintained after the multivariate analysis. The logistic regression model was statistically significant, *X*^2^ = 39.973, *p* < 0.001. The model explained 43.5% (Nagelkerke’s R2) of the variance in 10-year persistence and correctly classified 83.2% of cases. The sensitivity, specificity, positive predictive value and negative predictive value of the model were 56.7%, 92.8%, 56.7% and 92.8%, respectively. Of the six predictor variables, only four were statistically significant, male sex (OR = 7.299, 95% CI: 2.067–25.773, *p* = 0.002), *LMO4* rs9833320 (OR = 4.484, 95% CI: 1.542–13.042, *p* = 0.006), *IL20RA* rs11678460 (OR = 0.120, 95% CI: 0.024–0.597, *p* = 0.010) and biological treatment (OR = 5.456, 95% CI: 1.571–18.952, *p* = 0.008).

The presence of the mutated allele (T) at rs983332 increased the likelihood of persistence at 10 years (β = 1.501), whereas the presence of the mutated allele (C) at rs11678463 was associated with non-persistence (β = −2.117). Similarly, persistence at 10 years was more likely in men compared to women (β = 1.988) and in patients treated with adalimumab, infliximab and ustekinumab compared with those treated with etanercept (β = 1.697).

## 4. Discussion

Numerous articles in the current literature report on the efficacy and short-term persistence of the different biologic agents available for the treatment of psoriasis. However, only one study also evaluating 10-year persistence was found, and only of adalimumab and infliximab [7]. Furthermore, the data in this present work were divided according to whether the drug was given as a first, second or third choice, since there may be greater efficacy in patients who are being treated with a biologic for the first time than in those who have previously failed with this type of therapy [10,11]. In the case of adalimumab, there was a statistically significant finding of lower response as a second or third option, and there was also a trend for infliximab and ustekinumab, although in this case it was not significant.

The persistence rate was 41.5% for adalimumab, 28% for infliximab and 34.8% for ustekinumab. The confidence intervals between them overlapped, so there were no significant differences, as shown in other studies [12]. However, when the three drugs mentioned were compared with etanercept (9.3% persistence), there was a significant difference. In the work of Onsun et al. [7], similar results to those presented here were observed: adalimumab persistence at 10 years (25.1%) was higher than that of infliximab (17%). However, in a different study, differences between adalimumab and ustekinumab persistence reached significance, with the former being more effective than the latter [13]. The results of this study may be biased because it is an evaluation of persistence at 3 years [13] and not at 10 years as in our study.

The most common reason for discontinuation in the first, second and third lines of the four drugs was insufficient efficacy [14], although a non-significant trend was observed. The results of this study reflected homogeneous discontinuation rates due to insufficient efficacy among the four drugs (51.2% adalimumab, 57.4% etanercept, 52% infliximab and 34.8% ustekinumab). However, in the other study with a 10-year follow-up, the discontinuation rates (22.8% for adalimumab and 13% for infliximab) were lower than in our research, differences probably caused by the distinction between insufficient primary and secondary efficacy [7]. Furthermore, it should be noted that in our study, insufficient efficacy was assessed according to the criteria of the physician or even the patient himself.

With regard to the described population variables (see Table 1), those that tend to modify the course of the disease and the efficacy of biologic therapy (sex, smoking, age at diagnosis of psoriasis, PASI at the start of treatment or psoriatic arthritis) were collected [15]. There was a trend towards lower persistence in patients who were smokers and in those with psoriatic arthritis or with cardiovascular risk factors (see Table 4) [16]. This result may be related to the lack of adherence to therapy in the aforementioned pathologies and habits, which may be associated with a lack of adherence to biologic treatments and, therefore, lower persistence. The female sex variable, on the other hand, showed a significant difference with a lower persistence at 10 years, contrary to what was observed in the other 10-year persistence study [7], which did not find a significant difference between the sexes. There may be less persistence in women because they received etanercept more frequently, but there must also be some influence of sex because the effect is maintained in the multivariate analysis.

Another reason for discontinuation was the occurrence of adverse events. Among these four drugs, ustekinumab is the one with the fewest adverse events according to the literature [17], a fact that agrees with the figures reflected in our study in the three lines of treatment with this drug. Etanercept was the drug with higher incidence of adverse reactions in the study in the three lines of treatment. However, a different study observed higher incidence of adverse reactions under adalimumab treatment [18].

Psoriatic arthritis was another important reason for discontinuation. The insufficient efficacy of biologics in the treatment of arthritis symptoms was highest, with ustekinumab at 8.7% in the first line and 6.5% in the second line. This result differs from the results obtained in another research study, where it was observed that patients treated with anti-IL12/23 drugs developed less psoriatic arthritis than those treated with anti-TNF [19].

It was also observed (Table 4) that in the case of etanercept and infliximab, which were the drugs with the lowest persistence at 10 years (9.3% and 28%, respectively), there was a higher PASI than in patients treated with the other drugs (23.5 ± 12.4 in etanercept and 27.3 ± 12.6 in infliximab). In any case, there was no statistically significant relationship between initial PASI and persistence.

The treatment of some of the patients who persisted on any of the four drugs studied was modified with dose reductions due to good response to treatment. Adalimumab had the highest percentage of dose reductions among those who persisted, which is consistent with the literature [20]. In 82.4% of those who persisted in this study, the dosing interval was increased. Patients who persisted on infliximab and etanercept as first-line treatment did not have a dose reduction or interval increase.

Advancements in our understanding of the pathogenesis of psoriasis have facilitated the identification of numerous potential therapeutic targets, including anti-tumor necrosis factor (TNF)-α, anti-interleukin (IL)-17, anti-IL-12/23, and anti-IL-23 therapies [21,22]. These developments have significantly transformed the available treatment options for psoriasis. For example, the Group for Research and Assessment of Psoriasis and Psoriatic Arthritis (GRAPPAs) has developed treatment guidelines that encompass all of the aforementioned drugs. These guidelines are designed to optimize functional outcomes, improve quality of life and reduce complications [23]. Several studies highlight the high efficacy of guselkumab (anti-IL23) and secukinumab (anti-IL17) for the control of severe psoriasis, in addition to the control of psoriatic arthritis symptoms [13], which other older biologics do not resolve. However, there are still no studies evaluating long-term persistence with these more recently marketed drugs; it should be noted that they have only been on the market for a short time and results may vary over time. In addition, the price of the drug may be a drawback for public health care, since an injection of guselkumab costs about EUR 2500 every two months, compared to about EUR 2000 every two months for an older biologic such as adalimumab. In addition to being cheaper, it should be noted that there are more biosimilar drugs on the market that are older than guselkumab, which may reduce the price. However, treatment should be individualized for each patient depending on the history of biologics already used and the clinical presentation, as well as the resources available in each hospital.

The effect of genetic variation on treatment persistence at 10 years was studied. *LMO4* codes for a transcription factor, LIM-only protein 4, which regulates the proliferation and differentiation of epithelial cells during the process of embryogenesis. Furthermore, it has been demonstrated that this protein is expressed at a high level in skin lesions [24]. The presence of the mutated allele (T) in *LMO4* rs983332 was associated with persistence for treatment. Psoriasis is related to other pathologies such as rheumatoid arthritis [25], and in this pathology, this SNP has been found to be associated with a lower short-term response to anti-TNF therapy [26]. *IL20RA* codes for a receptor for IL20, a cytokine that may be involved in epidermal function and is associated with psoriasis in the Caucasian population [27]. In the case of *IL20RA* rs1167846, this is, to our knowledge, the first paper to report an association between this SNP and response to anti-TNF therapy. Further studies are needed to clarify the role of these SNPs in the response to anti-TNF drugs and their implication in treatment persistence.

## 5. Limitations

The limitations of the study include the small number of patients available and the loss of patients from the original database because they did not meet the inclusion criteria, as well as because of the rate of loss of follow-up. Furthermore, it would be optimal to have homogeneous groups within each treatment to minimize confounding factors. Also, the fact that this is a retrospective observational study allowed the evaluation of the efficacy and persistence of the drugs, but not as objectively as could be assessed in a clinical trial or a prospective observational follow-up study.

## 6. Conclusions

The 10-year persistence of first-line treatment was 25.9%. The persistence rates in adalimumab, etanercept, ustekinumab and infliximab were 41.5%, 9.3%, 28% and 34.8%, respectively. Furthermore, there was a decrease in efficacy when the drug was administrated as a second- or third-line treatment. The most common reason for discontinuation was insufficient efficacy for the four biologics (51%), although adverse reactions to etanercept (9.3%) and lack of improvement in psoriatic arthritis were also relevant. Dose reductions were applied to 82.4% of patients who persisted on adalimumab and 37.5% of patients who persisted on ustekinumab. Overall, 10-year persistence was related to sex (higher in men), biologic drugs and polymorphisms in *IL20RA* (rs1167846) and *LMO4* (rs983332).

## Figures and Tables

**Table 1:** Description of the study population and distribution of first-line biological treatment to patients.

	Total (*n* = 143)	Women (*n* = 61)	Men (*n* = 82)	*p*
Age at diagnosis (years)	24.4 ± 15	25.1 ± 15	23.8 ± 15.1	0.250
Age at the start of biological treatment (years)	44.7 ± 15.1	44.9 ± 17.1	44.4 ± 15.1	0.180
Weight (kg)	78.7 ± 16	71.3 ± 16	83.6 ± 16	0.018
Basal PASI	22 ± 12.4	20.3 ± 12.4	23.3 ± 12.4	0.700
Psoriatic arthritis	55 (38.5%)	28 (45.9%)	27 (32.9%)	0.115
Smokers	32 (22.4%)	9 (14.8%)	23 (28%)	0.059
Cardiovascular risk factors				
Arterial hypertension	34 (23.8%)	11 (18%)	23 (28%)	0.186
Dyslipemia	40 (28%)	18 (29.5%)	22 (26.8%)	0.938
Diabetes Mellitus	17 (11.9%)	6 (9.8%)	11 (13.4%)	0.475
First-line biological treatment				
Adalimumab	41 (28.7%)	16 (26.2%)	25 (30.5%)	0.578
Etanercept	54 (37.8%)	29 (47.5%)	25 (30.5%)	0.037
Infliximab	25 (17.5%)	8 (13.1%)	17 (20.7%)	0.143
Ustekinumab	23 (16.1%)	8 (13.1%)	15 (18.3%)	0.405

Results are presented in the table as “mean ± standard deviation” or “*n* (percentage)”. PASI = Psoriasis Area and Severity Index.

**Table 2:** Assessment of persistence and reasons for discontinuation of adalimumab, etanercept, infliximab and ustekinumab in first-line therapy.

	Total (*n* = 143)	ADA (*n* = 41)	ETN (*n* = 54)	INF (*n* = 25)	UST (*n* = 23)
10-year persistence	37 (25.9%)	17 (41.5%)	5 (9.3%)	7 (28%)	8 (34.8%)
Reasons for discontinuation					
Insufficient efficacy	73 (51%)	21 (51.2%)	31 (57.4%)	13 (52%)	8 (34.8%)
Patient choice	7 (4.9%)	0	5 (9.3%)	0	2 (8.7%)
Lack of control of psoriatic arthritis	3 (2%)	0	1 (1.9%)	0	2 (8.7%)
Adverse reactions	8 (5.6%)	1 (2.4%)	5 (9.3%)	2 (8%)	0
Pregnancy	1 (0.7%)	0	1 (1.9%)	0	0
Precautionary suspension due to other pathology *	3 (2%)	0	1 (1.9%)	1 (4%)	1 (4.4%)
Good response	2 (2%)	0	2 (3.8%)	0	0
Loss to follow-up	9 (6.3%)	2 (4.8%)	3 (5.7%)	2 (8%)	2 (8.7%)

Results are presented in the table as “*n* (percentage)”. ADA: adalimumab; ETN: etanercept; INF: infliximab; UST: ustekinumab. * Renal cancer surgery, aortic aneurysm, tuberculous infection, pancreatic cancer diagnosis and severe COVID admission.

**Table 3:** Comparison of population variables with the persistence of the four drugs under study.

	ADA (*n* = 41)	ETN (*n* = 54)	INF (*n* = 25)	UST (*n* = 23)	*p*
Age at start (years)	43.1 ± 13.8	44.7 ± 16.7	41.7 ± 11.6	49.2 ± 14	0.799
Weight (kg)	80.7 ± 16.3	75.1 ± 15.9	75.9 ± 16.3	85.3 ± 16	0.130
Sex (women)	39% (16)	53.7% (29)	32% (8)	34.8% (8)	0.199
Basal PASI	18.6 ± 12.4	23.5 ± 12.4	27.3 ± 12.6	19.9 ± 12.5	0.154
Psoriatic arthritis	39% (16)	40.7% (22)	32% (8)	39.1% (9)	0.903
10-year persistence	41.5% (17)	9.3% (5)	28% (7)	34.8% (8)	0.002
Dose reduction in those who persisted	82.4% (14)	0	0	37.5% (3)	0.006
Discontinued due to insufficient efficacy	51.2% (21)	57.4% (31)	52% (13)	34.8% (8)	0.094

Results are presented in the table as “mean ± standard deviation” or “percentage (*n*)”. ADA: adalimumab; ETN: etanercept; INF: infliximab; UST: ustekinumab. PASI = Psoriasis Area and Severity Index.

**Table 4:** Comparison of patients who persisted 10 years with those who did not persist 10 years.

	Persist 10 Years (*n* = 37)	No Persist (*n* = 106)	*p*
Age at diagnosis (years)	23.2 ± 4.3	24.1 ± 14	0.540
Age at start (years)	43.6 ± 10.7	44.4 ± 16.16	0.283
Weight (kg)	82.8 ± 15.8	76.3 ± 15.8	0.193
Sex (women)	6 (16.2%)	55 (51.9%)	<0.001
Basal PASI	21.2 ± 12.3	18 ± 12.42	0.747
Psoriatic arthritis	10 (27%)	41 (38.7%)	0.063
Smokers	7 (18.9%)	24 (22.6%)	0.169
Arterial hypertension	6 (16.2%)	24 (22.6%)	0.09
Dyslipemia	8 (21.6%)	26 (24.5%)	0.184
Diabetes mellitus	1 (2.7%)	9 (8.5%)	0.112

Results are presented in the table as “mean ± standard deviation” or “*n* (percentage)”. PASI = Psoriasis Area and Severity Index.

**Table 5:** Significant SNPs in the univariate analysis at 10-year persistence.

Gene	SNP	Genotype	*n*	10-Year Persistence		*p*_uv_
				No	Yes	
*LMO4*	rs983332	G/G	70	57 (81.4%)	13 (18.6%)	0.014
		G/T	39	24 (61.5%)	15 (38.5%)	
		T/T	5	2 (40.0%)	3 (60.0%)	
		Total	114	83	31	
*IL20RA*	rs1342642	G/G	59	46 (78.0%)	13 (22.0%)	0.040
		G/A	40	31 (77.5%)	9 (22.5%)	
		A/A	15	7 (46.7%)	8 (53.3%)	
		Total	114	84	30	
	rs1167846	T/T	13	5 (38.5%)	8 (61.5%)	0.013
		T/C	48	38 (79.2%)	10 (20.8%)	
		C/C	54	41 (75.9%)	13 (24.1%)	
		Total	115	84	31	
	rs1184860	C/C	15	7 (46.7%)	8 (53.3%)	0.040
		C/T	50	40 (80.0%)	10 (20.0%)	
		T/T	50	37 (74.0%)	13 (26.0%)	
		Total	115	84	31	

*LMO4*: LIM Domain Only 4. *IL20RA*: Interleukin 20 Receptor Subunit Alpha. Underlined: *p*_mv_ < 0.05 in multivariate analysis.

## Data Availability

Data belonging to the study may be accessible upon reasonable request to the corresponding authors.

## Supplementary Materials

The following supporting information can be downloaded at https://www.mdpi.com/article/10.3390/biomedicines13010005/s1. Table S1: Study of the population, persistence and reasons for discontinuation of patients who received adalimumab as their first, second or third biologic treatment. Table S2: Study of the population, persistence and reasons for discontinuation of patients who received etanercept as their first, second or third biologic treatment. Table S3: Study of the population, persistence and reasons for discontinuation of patients who received infliximab as their first, second or third biologic treatment. Table S4: Study of the population, persistence and reasons for discontinuation of patients who received ustekinumab as their first, second or third biologic treatment.
