# Supplementary material for: Ten-Year Persistence of Biologic Drugs in Psoriasis and Its Relationship with Pharmacogenetic Biomarkers
Source: Biomedicines. 2024 Dec 24;13(1):5. doi: 10.3390/biomedicines13010005 (PMC11762171; doi:10.3390/biomedicines13010005)
Supplement: Supplementary file 1 [file biomedicines-13-00005-s001.zip › biomedicines-3391984-supplementary.pdf]

Supplementary Table S1. Study of the population, persistence and reasons for discontinuation of patients who received adalimumab as their first, second or third biologic treatment.

	**First-line treatment**			**Second- and third-line treatment**		
Sex	**Total (n=41)**	**Women (n=16)**	**Men (n=25)**	**Total (n=31)**	**Women (n=16)**	**Men (n=15)**
Basal PASI	18.6 ± 12.4	17.1 ± 12.7	19.2 ± 12.5	21.1 ± 12.9	18.7 ± 11.5	23.8 ± 14.3
10-years persistence	17 (41.5%)	2 (12,5%)	15 (60%)	5 (16.1%)	3 (18.8%)	2 (13.3%)
Reasons for discontinuation						
Insufficient efficacy	21 (51.2%)	11 (68.8%)	10 (40%)	17 (54,8%)	10 (63%)	7 (46.7%)
Patient choice	0	0	0	0	0	0
Lack of control of psoriatic arthritis	0	0	0	2 (6.5%)	0	2 (13.3%)
Adverse reactions	1 (2.4%)	1 (6.3%)	0	1 (3.3%)	0	1 (6.7%)
Pregnancy	0	0	0	0	0	0
Precautionary suspension due to other pathology*	0	0	0	1 (3.3%)	0	1 (6.7%)
Good response	0	0	0	1 (3.3%)	1 (6.3%)	0
Loss to follow-up	2 (4.8%)	2 (12.5%)	0	4 (13%)	2 (12.6%)	2 (13.3%)

Results are presented in the table as “mean±standard deviation” or “n (percentage)”. * tuberculous infection. PASI = Psoriasis Area and Severity Index.

Supplementary Table S2. Study of the population, persistence and reasons for discontinuation of patients who received etanercept as their first, second or third biologic treatment.

	**First-line treatment**			**Second- and third-line treatment**		
Sex	**Total (n=54)**	**Women (n=29)**	**Men (n=25)**	**Total (n=7*)**	**Women (n=4)**	**Men (n=3)**
Basal PASI	23.5 ± 12.4	22.2 ± 12.4	24.9 ± 12.4	29.3 ± 8.4	23.5 ± 3.6	38 ± 0
10-years persistence	5 (9.3%)	2 (6.9%)	3 (12%)	1 (14.3%)	1 (25%)	0
Reasons for discontinuation						
Insufficient efficacy	31 (57.4%)	19 (65.5%)	12 (48%)	0	0	0
Patient choice	5 (9.3%)	1 (3.5%)	4 (16%)	0	0	0
Lack of control of psoriatic arthritis	1 (1.9%)	1 (3.5%)	0	0	0	0
Adverse reactions	5 (9.3%)	2 (6.9%)	3 (12%)	0	0	0
Pregnancy	1 (1.9%)	1 (3.5%)	01 (3.5%)	0	0	0
Precautionary suspension due to other pathology**	1 (1.9%)	1 (3.5%)	0	0	0	0
Good response	2 (3.8%)	0	2 (8%)	0	0	0
Loss to follow-up	3 (5.7%)	2 (6.9%)	1 (4%)	0	0	0

Results are presented in the table as “mean±standard deviation” or “n (percentage)”. * One of the 7 patients received etanercept as a first treatment and discontinued it voluntarily, then received ustekinumab and years later returned to etanercept as a third line of treatment. ** Crohn's disease. PASI = Psoriasis Area and Severity Index.

Supplementary Table S3. Study of the population, persistence and reasons for discontinuation of patients who received infliximab as their first, second or third biologic treatment.

	**First-line treatment**			**Second- and third-line treatment**		
Sex	**Total (n=25)**	**Women (n=8)**	**Men (n=17)**	**Total (n=10)**	**Women (n=5)**	**Men (n=5)**
Basal PASI	27.3 ± 12.6	17.4 ± 12.3	33.8 ± 12.6	22.4 ± 12.6	30.8 ± 11.5	18.2 ± 10.4
10-years persistence	7 (28%)	0	7 (41.2%)	1 (10%)	1 (20%)	0
Reasons for discontinuation						
Insufficient efficacy	13 (52%)	7 (87.5%)	6 (35.3%)	7 (70%)	3 (60%)	4 (80%)
Patient choice	0	0	0	0	0	0
Lack of control of psoriatic arthritis	0	0	0	0	0	0
Adverse reactions	2 (8%)	1 (12.5%)	1 (14.3%)	1 (10%)	1 (20%)	0
Pregnancy	0	0	0	0	0	0
Precautionary suspension due to other pathology*	1 (4%)	0	1 (14.3%)	0	0	0
Good response	0	0	0	0	0	0
Loss to follow-up	2 (8%)	0	2 (28.6%)	1 (10%)	0	1 (20%)

Results are presented in the table as "mean±standard deviation" or "n (percentage)". *Renal cancer surgery. PASI = Psoriasis Area and Severity Index.

Supplementary Table S4. Study of the population, persistence and reasons for discontinuation of patients who received ustekinumab as their first, second or third biologic treatment.

	**First-line treatment**			**Second- and third-line treatment**		
Sex	**Total (n=23)**	**Women (n=8)**	**Men (n=15)**	**Total (n=44)**	**Women (n=20)**	**Men (n=24)**
Basal PASI	19.8 ± 12.5	20.3 ± 12.5	19.7 ± 12.5	17.2 ± 11.4	14 ± 7.6	20.8 ± 13.7
10-years persistence	8 (34.8%)	2 (25%)	6 (40%)	14 (31.8%)	11 (55%)	3 (12-5%)
Reasons for discontinuation						
Insufficient efficacy	8 (34.8%)	1 (12.5%)	7 (46.7%)	24 (54.5%)	8 (40%)	16 (67%)
Patient choice	2 (8.7%)	1 (12.5%)	1 (6.7%)	0	0	0
Lack of control of psoriatic arthritis	2 (8.7%)	2 (25%)	0	2 (4.5%)	0	2 (8.4%)
Adverse reactions	0	0	0	0	0	0
Pregnancy	0	0	0	0	0	0
Precautionary suspension due to other pathology*	1 (4.4%)	1 (12.5%)	0	1 (2.25%)	0	1 (4.2%)
Good response	0	0	0	2 (4.5%)	1 (5%)	1 (4.2%)
Loss to follow-up	2 (8.7%)	1 (12.5%)	1 (6.7%)	1 (2.25%)	0	1 (4.2%)

Results are presented in the table as “mean±standard deviation” or “n (percentage)”. * aortic aneurysm and severe COVID admission. PASI = Psoriasis Area and Severity Index.
